# Mendelizing all Components of a Pyramid of Three Yield QTL in Tomato

**DOI:** 10.3389/fpls.2015.01096

**Published:** 2015-12-15

**Authors:** Amit Gur, Dani Zamir

**Affiliations:** The Robert H. Smith Institute of Plant Sciences and Genetics in Agriculture, Faculty of Agriculture, The Hebrew University of JerusalemRehovot, Israel

**Keywords:** epistasis, mendelizing, yield, QTL, tomato, wild species

## Abstract

Molecular markers allowed breeders to mendelize quantitative trait loci (QTL) providing another demonstration that quantitative traits are governed by the same principles as single qualitative genes. This research extends the QTL analysis to two and three QTL and tests our ability to mendelize an oligogenic trait. In tomato, agricultural yield is determined by the weight of the fruits harvested per unit area and the total soluble solids (% Brix)–sugars and acids. The current study explores the segregation of multiple independent yield-related QTL that were identified and mapped using introgression lines (IL) of *Solanum pennellii* in cultivated processing tomato (*S. lycopersicum*). We screened 45 different double and triple IL-QTL combinations for agricultural yield, to identify QTL pyramids that behaved in an additive manner and were suitable substrate for mendelizing an oligogenic trait. A pyramid of three independent QTL that significantly improved Brix^∗^Yield (BXY - the soluble solids output per unit area) compared to M82 was selected. In the progenies of the tri-hybrid we bred using markers a nearly isogenic ‘immortalized F2.’ While the common mode of QTL–QTL interactions across the 45 IL-QTLs combinations was less than additive, the three QTLs in the selected triple-stack performed in an additive manner which made it an exceptional material for breeding. This study demonstrates that using the phenotypic effect of all 27 possible QTL-alleles combinations it is possible to make reliable predictions about the genotypes that will maximize the yield.

## Introduction

Yield is a key trait for commercially grown crop plants. The challenge in breeding and genetic analysis of yield is posed by the biological complexity of this trait, since yield reflects the cumulative effects of multiple factors over time and across plant organs and field environments. While the interaction of quantitative trait loci (QTL) with environmental conditions can be controlled in genetic studies, epistasis which is a major force shaping the phenotype remains a difficult component to quantitate (Carlborg and Haley, 2004; Mackay, 2014). The term ‘epistasis’ was coined approximately 100 years ago to describe the difference between predicted genetic segregation, based on the action of individual genes, and the observed product of a di-hybrid cross (Bateson, 1909). In the case of quantitative traits, epistasis refers to the deviation of a phenotype from its predicted value based on additivity between the effects of the underlying independent QTL (Carlborg and Haley, 2004). The role and importance of epistasis in the genetic architecture of quantitative traits remains controversial (Carlborg and Haley, 2004; Hill et al., 2008).

Recent advances in genomic technologies and computational capabilities have enabled more effective research to mendelize QTL and elucidate the role of epistasis in the evolution and genetic architecture of complex traits (Salvi and Tuberosa, 2005; Holland, 2007; Phillips, 2008; Mackay, 2014). However, the genetic dissection of interactions between QTLs remains a challenge and in many instances, requires the use of specific populations and appropriate designs to reduce genetic complexity and allow a focused and balanced analysis of interactions.

The nearly isogenic introgression lines (IL) population in tomato was developed 20 years ago (Eshed and Zamir, 1995) to effectively re-introduce unused genetic variation from wild species into the cultivated varieties and to facilitate efficient mapping of complex as well as simple traits that originate from the wild donor. The ILs consisted of marker-defined genomic segments transferred (through controlled crosses) from the drought-tolerant wild species *Solanum pennellii* into the genetic background of an elite inbred variety; M82 (Eshed and Zamir, 1995). The ILs population constitute a “genetic library,” where the whole wild species genome is divided into 76 lines, each carrying a single homozygous introgression. Implementation of this resource for QTL mapping exploits the nearly isogenic nature of the lines, such that any phenotypic difference between M82 and an IL, or the F1 cross of M82 with an IL (ILH), can be attributed to allelic effects within the corresponding *S. pennellii* genomic segments. The nearly isogenic nature of the IL population provides increased power and sensitivity for QTL mapping compared to whole-genome segregating populations (Keurentjes et al., 2007) and was extensively used over the last two decades to map QTLs for diverse traits (Lippman et al., 2007).

Epistatic relations among QTLs are often analyzed based on phenotyping whole genome segregating populations, such as F2, recombinant inbred lines (RIL), or double-haploids (DH) (Long et al., 1996; Li et al., 1997, 2014; Wentzell et al., 2007; Rowe et al., 2008; Buckler et al., 2009; Sandhu et al., 2014). The major weakness in this approach for estimating epistasis lies in the lack of statistical power to address digenic or higher order interactions due to the rarity of such genotypes in the population, the background effects of unlinked loci and the statistical problem of multiple tests (Mackay, 2014). Using structured crosses of NILs or ILs, epistasis can be tested in a nearly isogenic genetic background that includes only the target IL-QTL, which can provide better detection compared to RIL populations (Keurentjes et al., 2007; Melchinger et al., 2007; Reif et al., 2009) (Supplementary Figure S1). The focused nearly isogenic design is particularly beneficial for the analysis of QTL epistasis for low heritability traits, such as yield, where replicate trials are required to obtain solid estimates of the phenotype. This approach was used by Eshed and Zamir (1996) in their study of IL epistasis in tomato. The joint effect of IL-QTLs pairs was tested and compared to the individual effects of ILs. Out of 46 pairs of QTLs that had a significant effect in the same direction, 24 showed significant interaction (*P* < 0.05) and all were less than additive. A similar mode of epistasis was found for fruit quality QTLs in tomato (Causse et al., 2007). In autopolyploid sugarcane, sugar content showed diminishing return following an increase in favorable alleles doses (Ming et al., 2001). In mice, it was shown that the sum of the effects of individual QTLs is significantly greater than the phenotypic difference between the parental strains (Shao et al., 2008; Spiezio et al., 2012). Similar results were found for aggressive behavior in *Drosophila melanogaster* (Edwards and Mackay, 2009). It was suggested that the less-than-additive architecture could be a mechanism to ensure stability and canalization of phenotypes despite genetic and environmental disturbances (Gillespie and Turelli, 1989; Eshed and Zamir, 1996).

This study aimed to dissect and test all genotypes of a triple QTL stack that broke the less-than-additive trend and thus outperformed in our trials and produced high yield even when compared to leading varieties in the market (Gur and Zamir, 2004). This pyramid, constructed to include IL7-5-5, IL8-3, and IL9-2-5, was named IL789. It significantly and consistently improved tomato Brix and yield across diverse genetic backgrounds and environments, including drought conditions. The power of IL789 to break tomato yield prompted us to dissect it into its components and reassemble it in all possible genetic combinations. This exercise allowed us to extend the mendelian analysis of a single QTL to three QTL that segregate in the same tri-hybrid cross.

## Materials and Methods

### Plant Material

#### Introgression Lines

The parental lines of the IL population were the processing tomato, inbred variety M82 (*S. lycopersicum*), and the inbred accession of *S. pennellii* (LA716). The development and genetic characterization of the ILs population was previously described in details (Eshed and Zamir, 1995; Liu and Zamir, 1999). Further data are also available at the Solanaceae Genome Network (http://solgenomics.net/).

#### Pyramided Introgression Lines and Construction of 3-way ‘Immortalized F2’

**FIGURE 1:**
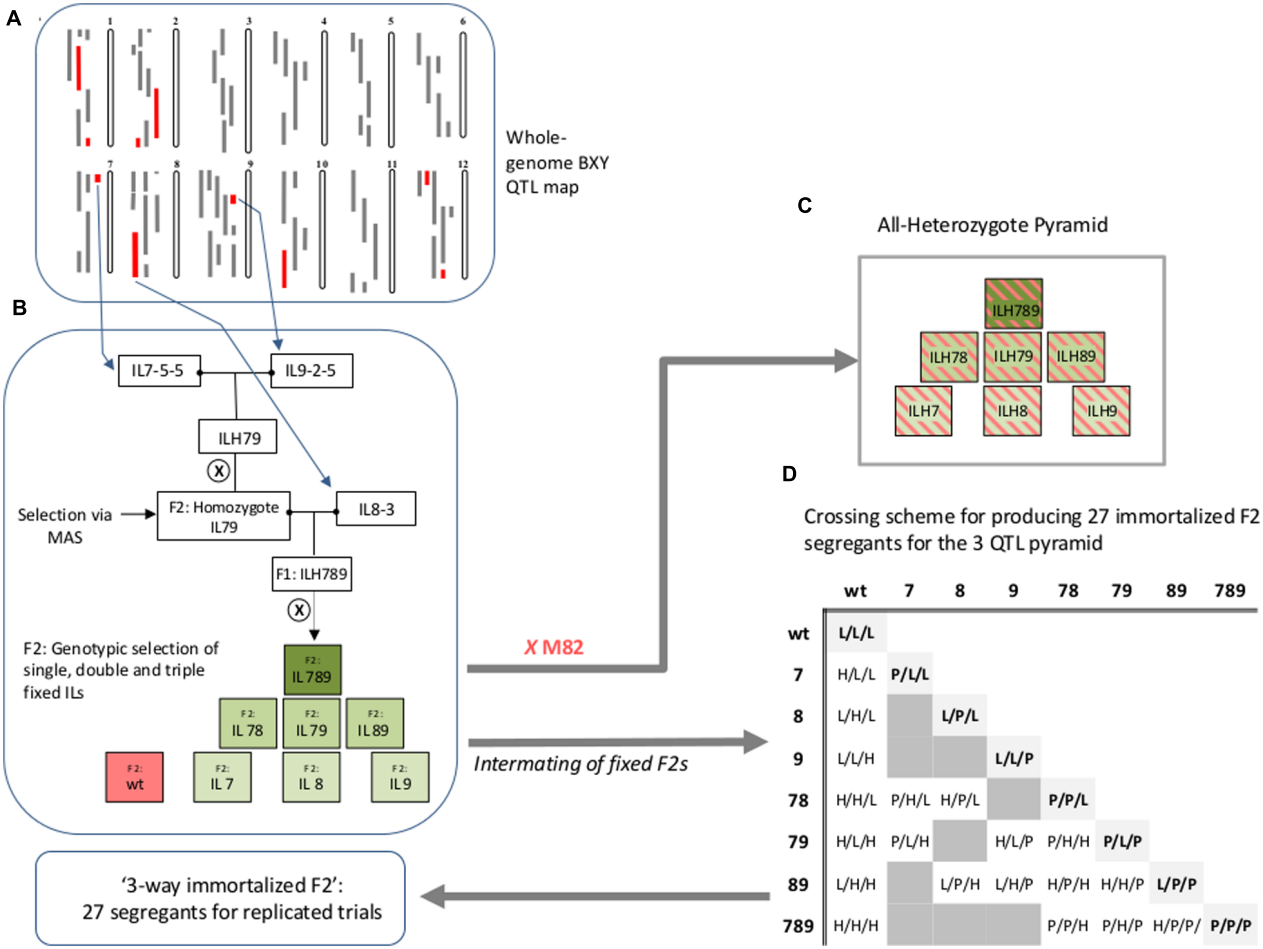
**Workflow for creation of IL789 pyramids and the ‘3-way Immortalized F2.’ (A)** BXY quantitative trait loci (QTL) map (Gur et al., 2011) used as the basis for selection of target IL-QTLs for pyramiding. The 12 tomato chromosomes are shown. Gray bars on the left of each chromosome are positions of introgressions at each of the *Solanum pennellii* ILs (Eshed and Zamir, 1995, http://solgenomics.net/). Red bars are introgression lines that showed significant increase in BXY compared to M82. **(B)** Description of the workflow for creation of the different QTL stacks. **(C)** The ‘All-Heterozygote’ pyramid, composed of single, double and triple introgression lines that were backcrossed to M82; all introgressions are present at the heterozygote state (ILH; IL-Hybrid). **(D)** Crossing scheme for producing 27 immortalized F2 segregants for the three QTL pyramid. Single, double and triple homozygote ILs were intermated to create the ‘3-way immortalized F2’. The numbers (7, 8, or 9) indicate the chromosome number and refer to the presence in homozygote state of *S. pennellii* introgressions IL7-5-5, IL8-3, and IL9-2-5, respectively. For the 27 genotypes at the ‘immortalized F2’ table; L = homozygote *lycopersicum*, H = Heterozygote, P = homozygote *pennellii*. Genotypes are expressed in the following order: IL7-5-5/IL8-3/IL9-2-5.

Pyramided genotypes were produced using crosses and marker-assisted selections (MASs) to identify and track the introgressed alleles. Independent ILs containing selected introgressions were crossed to produce double heterozygous ILs (ILHs). A set of 36 double ILHs was tested in the field in the first year. Best-performing entries were then self-pollinated and F2 plants homozygous for both introgressions were selected using markers. The fixed double ILs were then crossed to another selected single IL to produce triple ILHs. The new triple ILHs and selected double ILHs from the previous year, were tested under Dry field conditions (see below) in the second year. The selected triple-IL pyramid, IL789, was created by two rounds of crosses between the donor ILs (IL7-5-5, IL8-3, and IL9-2-5), followed by marker-assisted genotypic selection of desired combinations (**Figure 1B**). To explore the entire range of epistatic relations between the three single ILs, we produced an ‘immortalized 3-way F2’ that reflected all 27 possible genotypic combinations for segregation of three loci. An ‘immortalized F2’ is composed of fixed lines and structured crosses in such a way that provide large seed quantities for replicated trials using F2-like genotypes. Selected F2 plants [derived from self-pollination of the triple IL-hybrid (ILH789)] that were fixed for different combinations of the target introgressions were used for a structured crossing scheme to create the ‘immortalized 3-way F2’ (**Figure 1D**).

### Field Trials

Yield trials were carried out in the open-field experiment station in Akko, Israel. No specific permission is required to publish results of experiments conducted in the Akko Experiment Station that owns the fields where our trials were carried out. All the experiments were planted in the field in Akko in a randomized complete block design (RCBD) under two irrigation treatments (Wet and Dry) in 10 replications within each treatment as previously described (Gur and Zamir, 2004; Gur et al., 2011). Seedlings (35 days after sowing) were transplanted in the field with 50 cm between plants in a row and 2 m between rows (common experimental density of 1 plant per m^2^). Field was irrigated in the day of transplanting, with 30 m^3^ of water for every 1,000 m^2^ of field area. For the rest of the growing season, the Wet treatment was drip irrigated with 250 m^3^ per 1,000 m^2^, while no additional irrigation was applied to the Dry treatment.

### Phenotyping

Phenotyping was performed as previously described (Gur and Zamir, 2004; Gur et al., 2011). Experiments were harvested in a single harvest when on average 80–100% of the tomatoes were ripe (fully red). Red and green fruits were separated and weighed separately to estimate the variation in earliness of ripening between the tested genotypes. Plant vegetative weight (PW-Kg/m^2^) was determined by weighing only the vegetative tissue (after collecting the fruits) and after the roots were removed. Total fresh yield (TY-Kg/m^2^) per plant or plot reflect the sum of weights of the red and the green fruits. Average fruit weight (FW-g/fruit) was calculated from a random sample of 20 fruits per plant or plot. Concentrations of total soluble solids [BX, measured in degrees Brix (%)] were measured, using a hand refractometer (RFM-80 BS, ATAGO), from the juice of a random sample of ten fully ripe fruits per plant or 20 fruits per plot. Fruit number (FN-number of fruits/m^2^) was calculated as the ratio between TY (g/m^2^) and FW (g/fruit). The total sugar yield per plant was calculated as the BX^∗^TY (BXY-g sugar/plant).

### Genotyping

The ILs and derived populations were genotyped using Restriction Fragment Length Polymorphism (RFLP; Bernatzky and Tanksley, 1986) and PCR-based (CAPS) markers. For the MAS and stacking of target introgressions (as described in **Figure 1**), flanking markers from the ends of each introgression were used to track the alleles and ensure the integrity of the transmitted introgression. Exception is IL7-5-5 where inversion in this region between *S. penneellii* and S. *lycopersicum* (van der Knaap et al., 2004) is inhibiting recombinations and the use of single marker at the center of the introgression was sufficient. Markers that were used are: IL7-5-5: CT52. IL8-3: TG510, CT148, CT68. IL9-2-5: CP44, GP263. Detailed description of RFLP and CAPs markers positions and sequences are available at http://solgenomics.net/.

### Statistical Analyses

Statistical analyses were performed as previously described (Gur and Zamir, 2004; Gur et al., 2011) using the JMP V.8 software package (SAS Institute, Cary, NC, USA). The selection of IL-QTL for the 45 QTL stacks experiments was based on comparisons to the nearly isogenic recurrent parent; M82. Lines that showed a significant difference from M82 (*p* < 0.05, corrected for multiple comparisons using *Dunnet* test) were defined as possessing an IL-QTL. Less-than-additive hypothesis was tested by correlation analysis between expected and observed BXY values. Expected values were calculated as the sum of the single ILH effects based on the independent mean comparisons of each ILH to M82. QTL–QTL interactions were tested through ANOVA (using the “Fit model” function of JMP program) where each IL-QTL was tested as main effect and the double or triple QTL by QTL interactions were also tested as factors in the model. Comparisons between the pyramided combinations were performed using the “Fit Y by X” function of the JMP program followed by the “Compare all means” function that correct for multiple comparisons using the *Tukey–Kramer* test. Mode of inheritance parameters (a,d) for the three target IL-QTLs were calculated and previously described in details by Gur et al. (2011).

All the raw data for the presented experiments is available for download from Phenome Networks Project Unity http://unity.phenome-networks.com/ in a folder entitled “Dissecting 3-way Pyramid”.

## Results

### Less-than-additive is the Prevalent Mode of Interaction between Yield QTL in the IL Population

**FIGURE 2:**
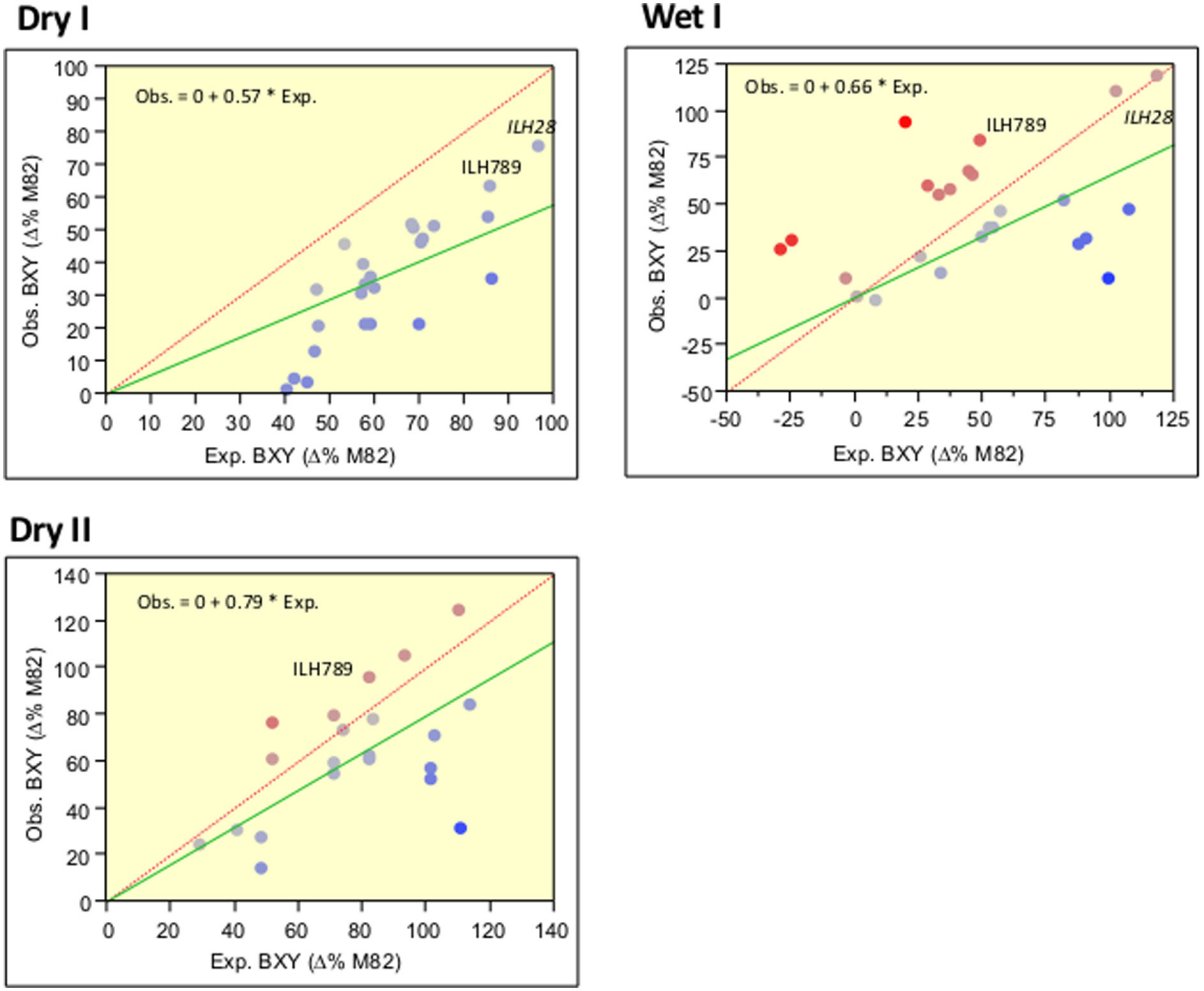
**Correlations between expected and observed BXY across 45 double and triple hybrids in the wet and dry fields over 2 years**. Expected (*X* axis) and observed (*Y* axis) BXY values are presented for double and triple ILHs that resulted from crosses between single or double ILs. BXY values are presented as percent difference from M82. Expected values were calculated as the sum of the single ILH effects, assuming complete additivity. The dashed red line represents the expected regression line where X = Y and intercept is 0,0. The green line represents the observed regression line, with the intercept constrained to 0,0, which is the value of M82. Equations for the observed by expected regression lines are presented at the left upper corner of each box. Slopes of all regression lines are significantly lower than 1. The Red-to-Blue scale reflects the deviation from the X = Y line.

To identify yield-enhancing QTL stacks, we screened, over a period of 2 years, 45 double and triple IL combinations and compared them to M82 and the single ILs. This survey included selected genotypes that were tested as ILHs and were compared to their double ILH combinations. By comparing the yield of the independent ILHs with the values of the double ILHs, we were able to determine whether there is a deviation from additivity indicative of epistasis. Our results were consistent with those of the past (Eshed and Zamir, 1996) and overall, there was a strong trend for the less-than-additive model. This trend can be viewed in **Figure 2**, where the slopes of the regression lines (green), whose intercepts were constrained to zero (which represents the M82), were significantly lower than 1, which is the slope of the expected regression line when assuming complete additivity. Each point on this figure represents a double or triple-ILH, where the predicted BXY (based on the sum of the effects of independent ILHs) is plotted against the observed BXY. To improve the confidence of this analysis aimed at identifying non-epistatic QTL combinations, we conducted the trial over two irrigation regimes: irrigated (Wet) and non-irrigated (Dry) trials, performed at the same location. The less-than-additive pattern seemed to be more prominent under drought stress, while under the Wet treatment, there was more variation but the same pattern of diminishing returns was evident. Despite the general less-than-additive trend, we identified few combinations that showed additivity, where the two with the most dramatic and consistent effect were the double-stack ILH28 and the triple stack IL789, that are described below.

### Analysis of Epistasis in the ILH28 Double Hybrid

**FIGURE 3:**
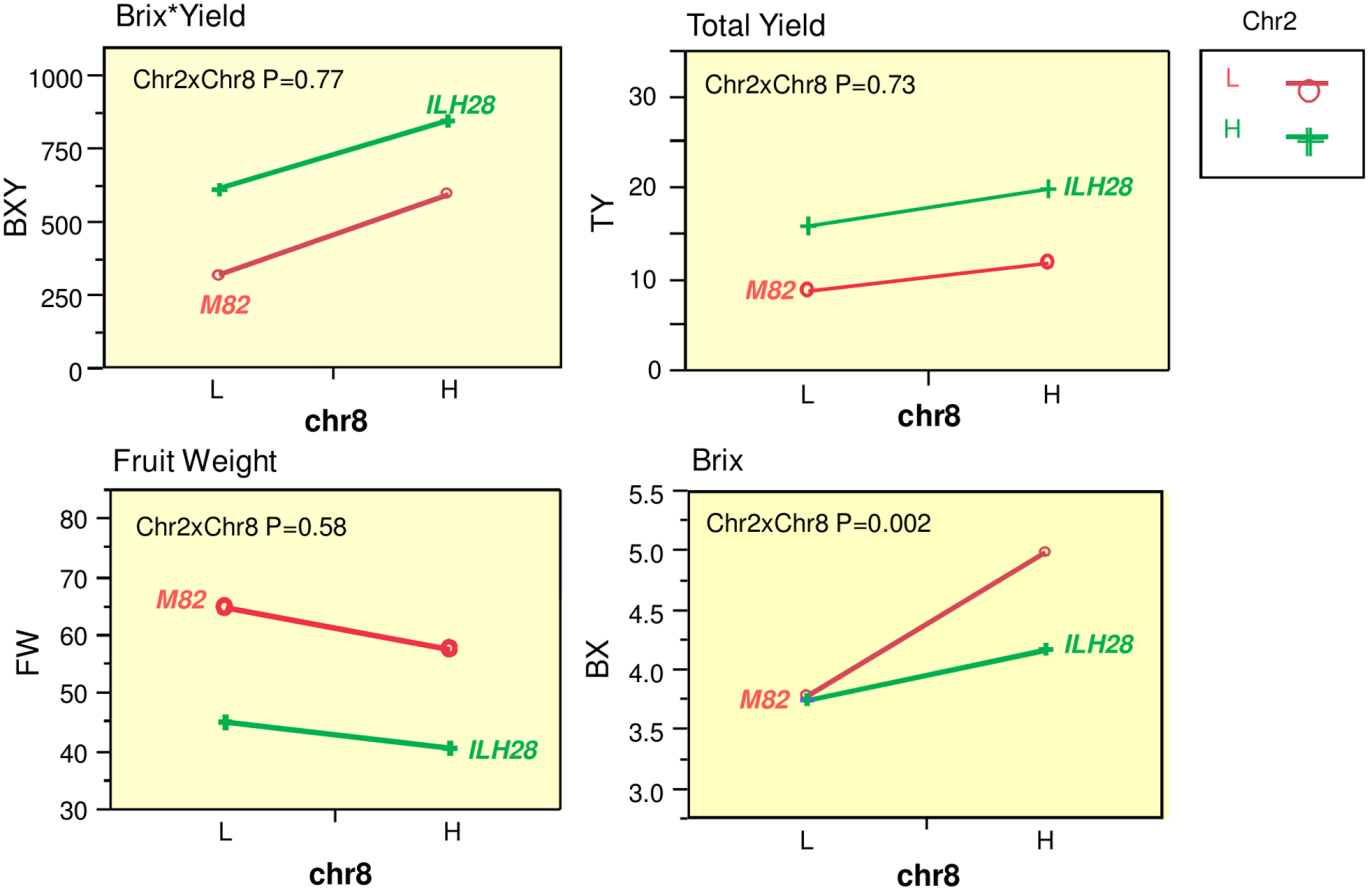
**Interaction plots for yield components between the underlying QTLs at the ILH28 double-stack**. Two-locus genotypic effects for ILH2-5, ILH8-3, and the double introgression hybrid ILH28. Presented are plots for Brix^∗^Yield (BXY; g sugar/m^*2*^), Total Yield (TY; Kg/m^*2*^), Fruit Weight (FW; g), Brix (BX; %). *X* axis (Chr8) are genotypes at IL8-3. The red lines represent genotypes homozygous for the *S*. *lycopersicum* allele at IL2-5, the green lines represent genotypes heterozygous at IL2-5. L = homozygote *S. lycopersicum* allele. H = Heterozygote. Chr2 reffer to IL2-5, Chr8 refer to IL8-3. Chr2xChr8 reflects the interaction as calculated from a two-way ANOVA.

A QTL combination that was interesting and showed significant double-QTL additivity was the double hybrid ILH28 (**Figure 2**), built from the cross between IL8-3 and IL2-5. IL8-3 and IL2-5 are two lines that consistently improved yield components and both showed a heterotic mode of inheritance, as the ILHs were superior to both the IL and to M82. IL2-5 contains introgression at the distal part of the long arm of chromosome 2, covering the position of the fruit-size gene *fw.2.2* (Frary et al., 2000). The wild allele of *fw.2.2* is associated with a very significant additive fruit size-decreasing effect and therefore, hybrids with IL2-5 on the processing tomato background, produce ‘midi-plum’-type tomatoes that are ∼30% smaller than the common processing tomato fruit. These QTLs were tested for their combining pattern and epistatic relations over two seasons. The double ILH was tested in yield trials alongside the single ILHs and M82. Results from two seasons showed strong consistency and no genotype^∗^year interactions were detected in a two-way ANOVA. Therefore, we pooled the data from both years and analyzed it together. **Table 1** summarizes the effects of the two QTLs; both ILH2-5 and ILH8-3 QTLs increased total yield (TY) by 78 and 33%, respectively, compared to M82. This heterotic yield increase was associated with a 31% decrease in fruit size for ILH2-5, and a non-significant 11% reduction in fruit size in ILH8-3. These results imply that yield increase of both QTLs is caused by an increase in the fruit number per plant. Brix of ILH2-5 was not different from that of M82, while ILH8-3 had a 33% higher Brix compared to M82. The BXY was increased by 91 and 86% in ILH2-5 and ILH8-3, respectively. To test the interaction between these two BXY-improving QTLs, we performed a two-way ANOVA and tested the QTL-QTL interactions for the above yield components (**Figure 3**). For the ultimate output of processing tomatoes, i.e., sugar production per unit area (BXY), QTLs performed in an additive manner and the interaction was not significant (*p* = 0.77). For TY, the expected performance of the double hybrid, as determined from the sum of independent effects, was 111% compared to M82, while the observed performance of the double hybrid was a 123% increase (**Table 1**). However, this more-than-additive pattern was not significant (*p* = 0.73), and was supportive of complete additivity. The only measured trait that showed interaction between the QTLs was Brix (*p* = 0.002, **Figure 3**), as reflected by the fact that ILH28, the double ILH, increased Brix by 10% compared to M82, which is significantly different from the expected effect based on the additive model, which was 32%.

**Table 1:** Effects of ILH2-5, ILH8-3, and the double ILH28 on yield-related traits.

		ILH2-5	ILH8-3	ILH28
TY	L	9.0	9.0	9.0
	H	16.0	12.0	20.1
	Δ% (H-L)	78%^∗∗^	33%	123%^∗∗^
BXY	L	323	323	323
	H	617	600	850
	Δ% (H-L)	91%^∗∗^	86%^∗∗^	163%^∗∗^
FW	L	64.8	64.8	64.8
	H	45.0	57.8	40.7
	Δ% (H-L)	-31%^∗∗^	-11%^∗^	-37%^∗∗^
BX	L	3.8	3.8	3.8
	H	3.8	5.0	4.17
	Δ% (H-L)	-1%	33%^∗∗^	10%^∗^

*TY, Total Yield (kg/m^2^); BXY, Brix^∗^Yield (g Sugar/m^2^); FW, Fruit Weight (g); BX, Brix (%). L = homozygote *S. lycopersicum* allele. H = Heterozygote. Δ% (H-L) = the phenotypic difference (in %) between the H genotype and the L genotype ^∗^*P* < 0.05, ^∗∗^*P* < 0.01*.

### The IL789 Pyramid

#### Independent Effects of the Individual ILs

**FIGURE 4:**
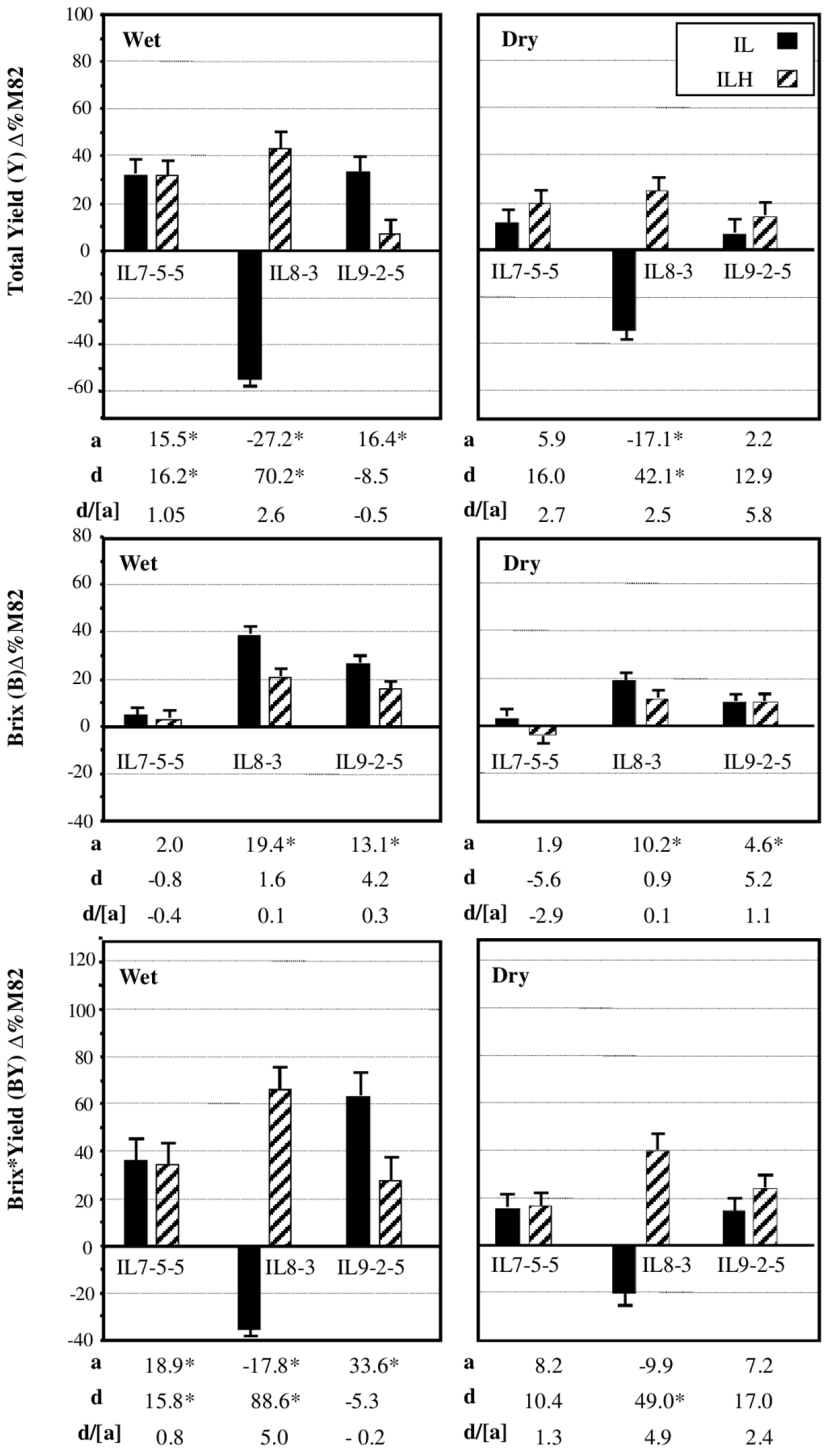
**Phenotypic effects of IL 7-5-5, IL8-3 and IL9-2-5 under Dry and Wet field conditions**. Introgression lines IL7-5-5, IL8-3, and IL9-2-5 were compared to M82 (data are presented as percent difference from M82) in homozygous (IL) and heterozygous (ILH) states, under Wet and Dry field condition. The bars represent total yield (TY), Brix (BX), and Brix^∗^Yield (BXY) least-square means (± standard error) from three growing seasons; these data were pooled, since no season × genotype interactions were found. The baseline represents M82, where the mean BXY values of M82 from the three seasons was 353 g/m^2^ in the irrigated treatment and 184 g/m^2^ in the dry treatment. The additive effect *(a)* is half of the difference between each IL and M82. The dominance deviation *(d)* is the difference between ILH and the mid-value of its parents. Values marked by an asterisk are significant changes from baseline (*p* < 0.05). All experiments were transplanted in a randomized block design with 10–15 replications per entry.

We chose to focus on three independent introgressions, located on Chromosome 7 (IL7-5-5), Chromosome 8 (IL8-3), and Chromosome 9 (IL9-2-5), that affected the components of BXY and were described in details previously (Gur and Zamir, 2004). **Figure 4** summarizes the independent effects of these IL-QTLs on yield components from three growing seasons in wet and dry fields. These previously described results (Gur and Zamir, 2004) are briefly presented here as an integral reference for the current study. IL7-5-5 had dominant effect on yield, as both the homozygous IL and heterozygous ILH showed 30% higher yield, compared to M82, in wet field and a non-significant 12 and 22% increase in the dry fields. IL7-5-5 did not have an effect on Brix, but showed a dominant effect on BXY. IL8-3 in its homozygous form was significantly inferior to M82 for yield (-55 and -34% for the Wet and Dry treatments, respectively), but as heterozygote (ILH) showed 45 and 25% yield increase compared to M82 (Wet and Dry, respectively). This result reflects a significant overdominant effect for this introgression (*d/[a]* = 2.5); (Gur and Zamir, 2004; Gur et al., 2011). Brix of the heterozygote ILH8-3 was 20 and 10% higher than M82 in the wet and dry conditions, respectively. The increase in both yield and Brix of ILH8-3 led to the observed overdominant effect on BXY in both irrigation regimes (70 and 40% increases compared to M82 in the wet and dry fields, respectively; *d/[a]* = 5). The reduced yield of IL8-3 was possibly caused by a pleiotropic effect of a recessive slight leaf necrosis gene that had stronger effect under drought stress. IL9-2-5 had higher yield compared to M82 only at the homozygote state in the wet field. For both Brix and BXY IL9-2-5 showed additive mode of inheritance as the ILH was intermediate between M82 and the homozygous IL.

### Analysis of Main Effects and Interactions of the IL789 Pyramid

These three ILs were further crossed to create a triple-IL pyramid (designated IL789), which contained all the three introgressions (**Figure 1B**). It took two generations and one MAS step to get from the independent ILs to the triple IL-hybrid (ILH789), which was heterozygote at all three introgressions. In order to explore the epistatic relations among these three IL-QTL on a common genetic background, we produced lines that represented all the single, double and triple combinations of this pyramid (**Figure 1B**).

#### All-Heterozygote Pyramid

The first field experiment included fixed F3 lines (progenies from self-pollination of ILH789) that were homozygous for the different combinations, and their F1s with M82, where all introgressions were in heterozygous state (**Figure 1C**). This means that each line was either homozygote for all the relevant introgressions or heterozygote for all. These fixed F3 segregants and F1s, including an F3 line that had none of the introgressions (used as an internal genetic reference, corresponding to M82), were tested in the field, in a replicated trial, for their yield-related phenotypes. In order to test the interactions between the IL-QTL using these genotypes, we divided the experiment into two genetic groups: ‘all-homozygous’ pyramid (contained homozygotes at all introgressed loci) and ‘all-heterozygous’ pyramid (contained heterozygotes at all introgressed loci). Since the homozygous IL8-3 had a strong negative effect on yield, we used the ‘all-heterozygous’ pyramid only to evaluate the main effects and epistasis on total-yield (TY), Brix (BX) and Brix^∗^total yield (BXY). The main effect of each IL-QTL, along with the epistatic interactions between them, was tested in a three-way ANOVA, where the single introgressions and the double and triple interactions were tested as factors in the model. As shown in **Table 2**, none of the interactions were significant (at *p* < 0.05), while for each of the traits, at least two introgressions showed a significant effect. For BXY, the main effects of all the three introgressions were significant and none of the interactions were significant. These findings suggest an additive mode of action between these tested QTLs. This can also be demonstrated in a simple way, by comparing the expected BXY for ILH789 (based on complete additivity of the single introgression effects), which is 97% higher than M82, to the observed value, which was 120% higher than M82 (The difference between these values is not significant).

**Table 2:** Phenotypic effects of pyramided introgressions and the interactions between them in the All-Heterozygous pyramid.

Genetic factor	Effect (BXY)	Prob > | t|	Effect (TY)	Prob > | t|	Effect (BX)	Prob > | t|
**IL7-5-5**	**83.5**	**0.018**	**2.18**	**0.005**	-0.02	0.869
**IL8-3**	**132.5**	**0**	**1.64**	**0.033**	**0.64**	**0**
**IL9-2-5**	**85.1**	**0.016**	0.53	0.484	**0.58**	**0**
**Int-7^∗^8**	18.1	0.605	0.24	0.749	0.18	0.062
**Int-7^∗^9**	-61.6	0.08	-1.1	0.152	-0.07	0.499
**Int-8^∗^9**	-51.2	0.145	-0.9	0.241	-0.12	0.223
**Int-7^∗^8^∗^9**	54.6	0.12	1.29	0.093	-0.06	0.544

*BXY, Brix^∗^Yield (g Sugar/m^*2*^); TY, Total Yield (kg/m^*2*^); BX, Brix (%). For the main effects of each of the introgressions, the values are the difference between the M82 and the lines carrying the *S. pennellii* alleles in the heterozygous state. For the interactions, the effects are the deviation from the expected value, assuming no interaction. Bolded underlined values are significant at *p* < 0.05*

#### 3-Way ‘Immortalized F2’

**FIGURE 5:**
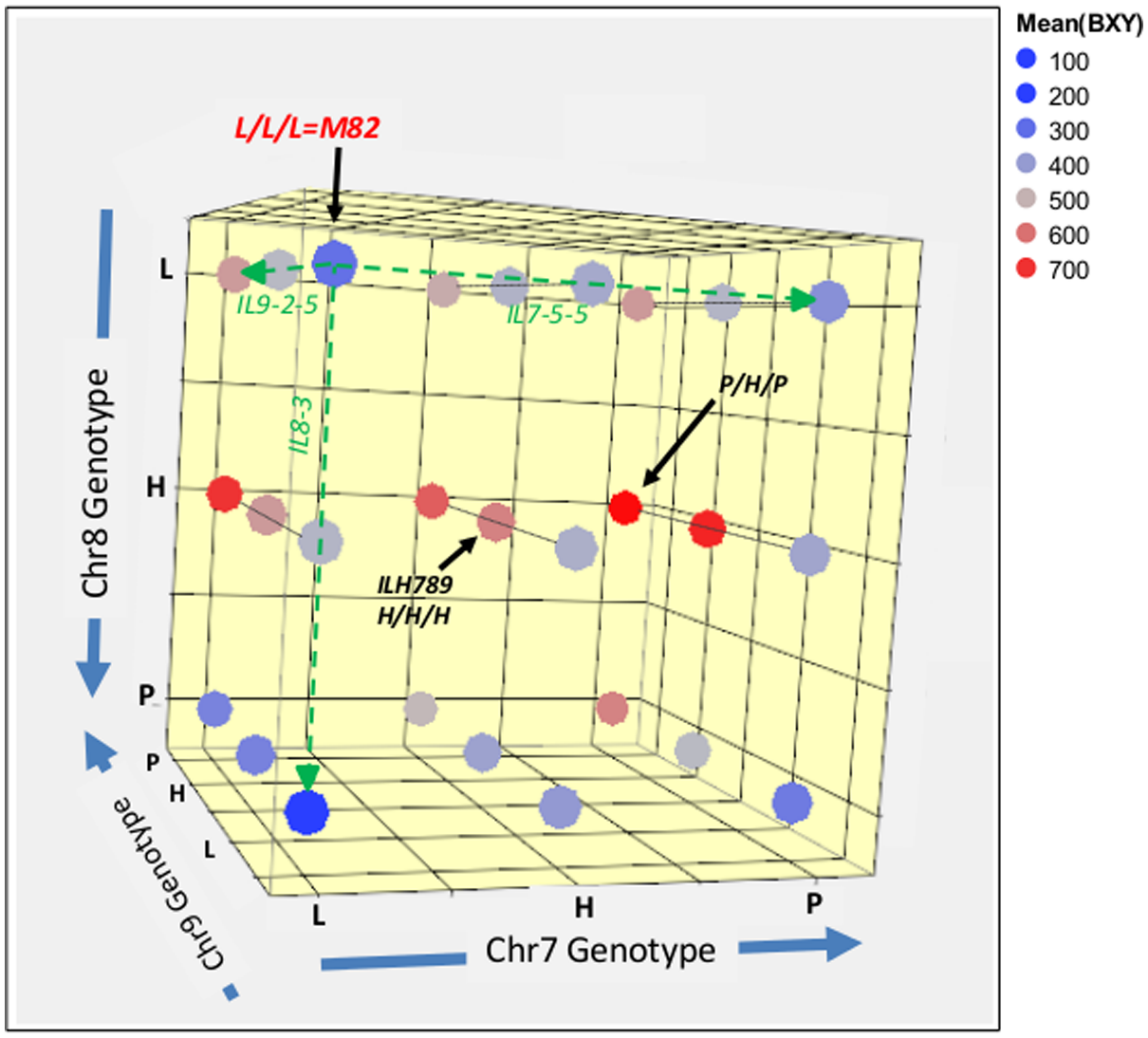
**3D genotype and BXY of the IL789 pyramid**. Each node represents a genotype from the 27 (3^∧3^) combinations. The different axes represent the different introgressions on chromosome 7, 8, and 9. L = homozygote *lycopersicum*, H = Heterozygote, P = homozygote *pennellii*. Colors reflect the mean BXY values for each genotype as calculated from 20 replicates of single plants. The green dashed line highlights single QTL effects.

In order to further explore the whole range of epistatic relations between these three IL-QTL, we produced a set of 27 genotypes that covered all the possible genetic combinations for these three unlinked introgressions. The common way to test a complete set of allelic interactions is to use an F2 population that segregates at the loci of interest and capture all possible genotypic combinations. The use of direct F2 segregants for the analysis of epistasis between QTLs for complex traits, such as yield, requires genotypic screening of large populations before planting, to ensure balanced representation of all genotypes and enough replicates to provide sufficient statistical power. To simplify this process, we decided to intermate between the available fixed single, double and triple pyramided ILs to create F1s that represented the 27 genotypic combinations and reflect F2 segregation (**Figure 1D**). These 27 genotypes, which can be regarded as an ‘immortalized F2,’ were then tested in a replicated field trial. The experiment included 27 genotypes × 20 replications, totaling 540 plants with a pre-defined genotype. Mean BXY values for each line from this trial were calculated and are projected in color-codes manner on the 3D genotypic matrix (**Figure 5**); a detailed multiple-range mean comparison between all 27 genotypes is available in Supplementary Table S1. **Figure 5** presents an overview of this experiment; the color transformation from the blue range to red/pink range on this figure, as we move between different nodes or along axes or surfaces from homozygote *S. lycopersicum* (L) to heterozygote (H) or homozygote *S*. *pennellii* (P), is an indication of the independent and additive effects.

The reference segregant from this population that is homozygous for the cultivated tomato alleles at all three QTL (L/L/L), is located in the left upper corner (dark blue; BXY = 310 g sugar/m^2^). As expected, this nearly isogenic reference was not significantly different from the common recurrent parent of the IL population, M82. The best-performing genotype in this pyramid was homozygote *S. pennellii* on chr7, heterozygote for chr8 (in consensus with the known heterotic effect of this QTL) and homozygote *S. pennellii* for chr9. This genotype is located on the right side of the middle surface (P/H/P; dark red, BXY = 759 g sugar/m^2^) and displayed a 145% higher BXY compared to the L/L/L segregant.

**FIGURE 6:**
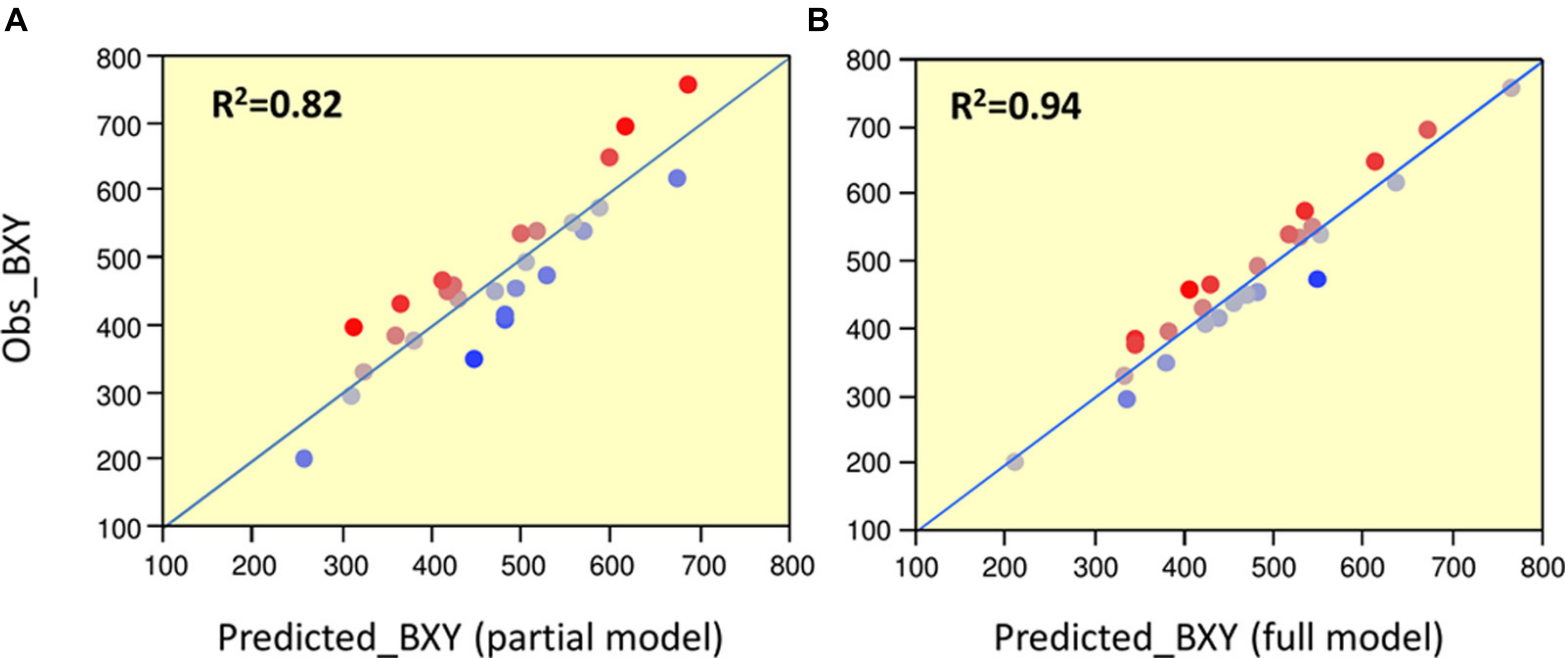
**Predicted vs. observed BXY for partial and complete models**. Regressions of predicted vs. observed BXY values for the 27 ‘immortalized F2’ genotypes. **(A)** Partial model: predicted values were calculated based on model with main effects alone. **(B)** Full model: predicted values were calculated based on model with main effects + QTL by QTL interactions. The Red-to-Blue scale reflects the deviation from the predicted regression line.

To determine significance of main effects and interactions, we performed a 3-way ANOVA. As shown in **Table 3**, all main effects of the individual QTLs were highly significant. Except for the interaction between Chr7 and Chr8 QTLs, that showed marginal significance (*p* = 0.0378), none of the other two or three-way interactions were significant at (*p* < 0.05). As presented earlier, IL7-5-5 seemed to have a dominant mode of action; IL9-2-5 was additive and IL8-3 had an over-dominant mode of action for BXY (**Figure 4**). These observations are consistent with current results that were obtained from the 27 genotypes from the dissected pyramid (**Table 3**). The BXY effects of each of the introgressions (as heterozygotes or homozygotes, in percent difference from M82) were calculated from the unified three-way ANOVA and confirmed the effects and mode of inheritance found for the independent ILs. Another way to test the impact of interactions in this QTL pyramid using this population, is by calculating predicted BXY values for each of the 27 segregants based on factorial model and Least-square mean estimates. Predicted BXY values were calculated in two ways: (1) using a model where only the main effects (of the three QTLs, without interactions) were entered and (2) using the complete model where double and triple interactions were also included as factors (**Figure 6**). The *R*^2^ from regression of predicted versus observed reflects the predictive value for each model. The partial model (assessing only the main effects; left box) showed an *R*^2^ of 0.82 and the complete model had an *R*^2^ of 0.94. While the complete model, as expected, was highly predictive, the partial model, that lacks interactions, still provided a very high *R*^2^, confirming the minimal impact epistasis has within this pyramid.

**Table 3:** 3-way Analysis of Variance (ANOVA) of BXY for the ‘immortalized F2’ from the IL789 pyramid.

						BXY (Δ%L/L/L)^c^	
Source^a^	Factor^b^	DF	Sum of squares	*F* Ratio	Prob > *F*	HETERO (H)	HOMO (P)
Chr7	Main	2	3750.65	5.13	0.0063	11	19
Chr8	Main	2	20041.7	27.43	<0.0001	29	-12
Chr9	Main	2	24767	33.90	<0.0001	32	51
Chr7 × Chr8	Interaction	4	3751.57	2.57	0.0378		
Chr7 × Chr9	Interaction	4	994.251	0.68	0.6059		
Chr8 × Chr9	Interaction	4	1658.79	1.14	0.3395		
Chr7 × Chr8 × Chr9	Interaction	8	3303.41	1.13	0.3418		

*^a^Factors tested in the model: main effects, two-way and three-way interactions. Chr7, Chr8, and Chr9 refer to IL7-5-5, IL8-3, and IL9-2-5, respectively. ^b^Category of the tested factors – main effect or interaction term. ^c^Phenotypic effects of the single introgressions in the heterozygous (H) and homozygous *pennellii* allele (P) states on Brix^∗^Yield (BXY). Effects are expressed as percent difference from the nearly isogenic internal reference line that is homozygote *lycopersicum* (L/L/L) at the three QTLs and was not statistically different from M82*.

## Discussion

Over the years, tens of trait loci from wild species donors have been mapped and introgressed into the cultivated tomato genome using MAS (Foolad, 2007). The *S. pennellii* IL population (Eshed and Zamir, 1995) was exploited to address two components related to tomato genetics and breeding: (1) introduction of unused favorable trait alleles that were neglected during tomato domestication and (2) creation of an effective framework for detection and mapping of QTL for complex traits, such as yield. The current study took a step forward by demonstrating the power of this framework for the identification of potential QTL stacks for yield improvement followed by detailed dissection of their combined performance. A three-step approach was taken: initially, using the IL population, individual yield-related QTLs were mapped and validated under Wet and Dry field conditions (Eshed and Zamir, 1995; Gur et al., 2011). The fact that the ILs were extensively characterized for yield components and comprehensive phenotypic data were captured into a unified database (Gur et al., 2004; Lippman et al., 2007) allowed us to generate a comprehensive profile for each IL and to identify QTLs that potentially improve yield components through diverse pathways. These lines served as candidates for pyramiding.

The combination of IL7-5-5, IL8-3, and IL9-2-5 is an example for this approach; the multi-year and multi-trait phenotypic characterization of these lines (and their F1s with M82; ILHs) indicated potential complementation and additive yield improvement. IL9-2-5 harbors at least two linked QTL that affect the components of BXY (Fridman et al., 2002). One of the QTLs is *Brix9-2-5*, which represents a single nucleotide polymorphism (SNP) that replaces an amino acid within the flower and fruit-specific apoplastic invertase (*LIN5*). The wild species allele increases sugar content of the fruit as a result of a modification of enzyme functions (Fridman et al., 2004). The other QTL within the IL9-2-5 introgression is seemingly associated with plant architecture and affects yield and Brix through modifications of sink-source ratios (Fridman et al., 2002). IL8-3 possesses at least one over-dominant yield-improving QTL. The yield increase is triggered by enhanced vigor that results in more flowers and fruits in plants carrying this introgression in a heterozygote state. IL8-3 also carries a recessive gene that causes slight leaf necrosis in plants homozygous for the *S*. *pennellii* allele. Through analysis of recombinant sublines of IL8-3, we were able to separate between the yield heterosis and the leaf necrosis linkage drag (unpublished data). Another interesting attribute of IL8-3 is a yield-increasing effect that acts through the roots, most likely triggered by a more vigorous root system. This root-specific QTL was identified and validated in grafting experiments where M82 grafted on IL8-3 produced higher yields than self-grafted and non-grafted M82, which were very similar to each other (Gur et al., 2011). The mode of action of IL7-5-5 requires further characterization but a recent study showed that this IL and IL8-3 both decreased cell number in the pericycle of the roots (Ron et al., 2013). From the multiple observations of IL7-5-5 in the field, we assume that the effect is related to improved fruit-setting under different environments, that may induce yield stability in genotypes carrying this QTL. The additivity within IL789 was confirmed in yield trials not only in the M82 background but also in commercially relevant hybrid combinations, where the IL789 pyramid significantly improved BXY across four different genetic backgrounds when compared to commercial hybrids (Gur and Zamir, 2004).

**FIGURE 7:**
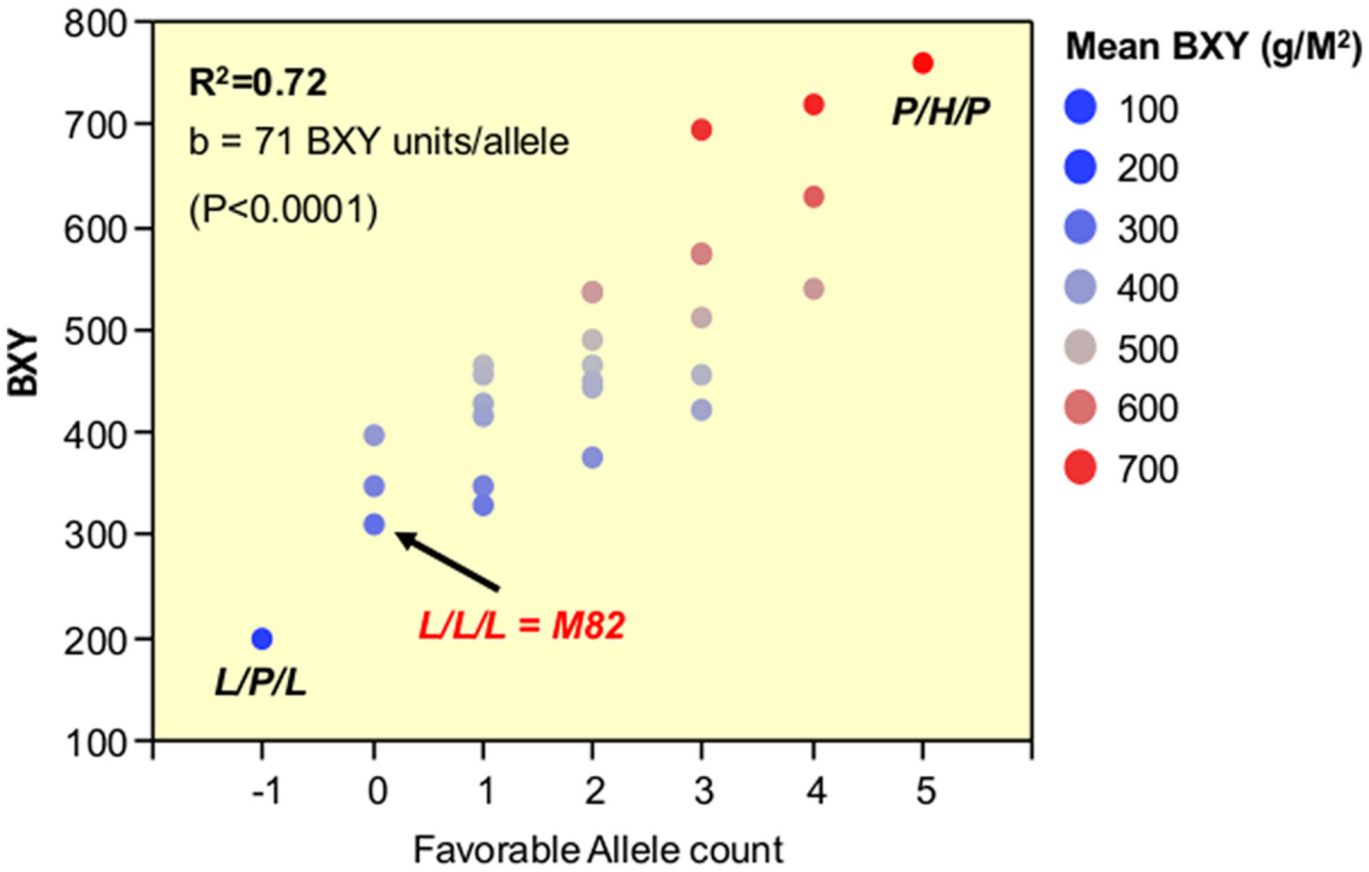
**Linear pyramiding effect: regression of favorable allele count against BXY, across 27 segregants from the IL789 pyramid**. The *X* axis is the favorable (*S. pennellii*) allele count. All wild alleles were counted in the same manner as +1, except for the *pennellii* allele at IL8-3 when present as a homozygote (P); this genotype was counted as -1 due to the negative impact of a recessive gene within this introgression causing partial sterility.

The third phase that is described in the current study dissected the triple-stack and reconstructed this pyramid to examine all possible interactions between the three independent QTLs. The nearly isogenic nature of the IL population allowed us to efficiently build a nearly isogenic triple-IL pyramid, break it down to its components through F2 segregation and then reconstruct it in the form of an ‘immortalized F2’ population that segregated to the three target QTLs only, thereby capturing all possible genotypic combinations. The fact that the tested ‘immortalized F2’ was generated from F2 segregants derived from the triple ILH provided further confidence in positioning the QTLs based on the defined introgression profile of the ILs and excluded the possibility of involvement of undetected small introgressions elsewhere. The 3-way nearly isogenic ‘immortalized F2’ design not only enabled testing of QTL interactions in a common genetic background (i.e., no other background segregation), it also allowed the testing of heterozygotic genotypes that are not present in other common segregating populations, such as RILs or DH. Based on this analysis, we were able to define the optimal allelic combination within the IL789 pyramid and to point to the best performing pyramided genotype. While in a previous study, the triple-stack was utilized in an ‘all-heterozygote’ form (ILH789-H/H/H; Gur and Zamir, 2004), we showed here that in accordance with the expected mode of inheritance of the independent QTLs (**Figure 4**, **Table 3**), the best performing pyramided genotype was homozygote for the *S. pennellii* allele at IL7-5-5, heterozygote for IL8-3 and homozygote for the *S*. *pennellii* allele at IL9-2-5 (P/H/P). This combination significantly improved BXY by 32% compared to the previously described ‘all-heterozygote’ genotype (**Figure 5**, Supplementary Table S1). To generalize this finding, the stacking impact can be best illustrated through the correlation between favorable allele count and BXY performance (**Figure 7**). Through a simplified model of equal effects of all participating favorable alleles (except for *S*. *pennellii* IL8-3 at the homozygote state), a significant linear regression between favorable allele count and BXY is shown. Despite the variation between different allelic combinations featuring the same favorable allele count, there was a significant linear increase in BXY as the number of favorable alleles increased from 0 to 5. The average contribution of wild favorable alleles in this stack was 71 g sugar per m^2^, corresponding to a 23% improvement compared to the internal reference that did not carry any *S. pennellii* allele.

At the most fundamental level, plant breeding is about stacking favorable allelic combinations to produce desired improved plant phenotypes. While such genetic improvements were achieved over thousands of years through direct phenotypic selections, in the past 20 years, it has become possible to genetically dissect traits and introduce breeding improvement by identifying and stacking discrete genetic components using linked molecular markers (Morgante and Salamini, 2003; Morrell et al., 2011). With the continuously increasing cost difference between genotyping and phenotyping, genotypic selection and indirect trait-allele-stacking in breeding is becoming the common practice for simple monogenic traits (Peleman and van der Voort, 2003). The challenge remains in implementation of such an approach for improvement of complex polygenic traits, such as yield, where multiple QTLs interact with each other and with the genetic background. Beside the complexity of identifying favorable discrete genetic components for yield, it is the interactions that limit the predictability and the effectiveness of this approach. An example for this limitation can be demonstrated through the varying yield effect of a mutation at the *SINGLE-FLOWER TRUSS* (*SFT*) gene (Krieger et al., 2010), where a dramatic heterotic yield improvement was shown for determinate tomatoes that were homozygotic for a recessive mutation at the *SELF-PRUNING* (*SP*) gene (*sp/sp*; Pnueli et al., 1998), while in indeterminate genetic backgrounds (*SP/+*), the *SFT* mutation did not show any effect on yield.

Exotic genetic libraries are a useful resource for crop improvement (Zamir, 2001). Identification and pyramiding of yield-related QTLs were performed in rice (Ashikari and Matsuoka, 2006; Zong et al., 2012) and tomato (Gur and Zamir, 2004). In the current study, we demonstrated a start-to-end pathway that uses an exotic nearly isogenic library to address yield improvement in tomato followed by thorough genetic dissection of epistasis between the three underlying pyramided loci. While nearly isogenic populations were shown to be a powerful tool for main-effect QTL mapping through the elimination of epistatic background effects (Eshed and Zamir, 1995; Keurentjes et al., 2007), they also provide advantages for analyzing epistasis through structured crosses (Eshed and Zamir, 1996; Melchinger et al., 2007; Reif et al., 2009). In this study we show that for an oligogenic trait it is possible to make reliable predictions about the genotypes that will maximize the phenotype and improve agricultural yield.

## Author Contributions

AG and DZ conceived and designed the experiments. AG performed the experiments. AG and DZ analyzed the data. AG and DZ wrote the paper.

## Conflict of Interest Statement

The authors declare that the research was conducted in the absence of any commercial or financial relationships that could be construed as a potential conflict of interest.
