# Supplementary material for: Mendelizing all Components of a Pyramid of Three Yield QTL in Tomato
Source: Front Plant Sci. 2015 Dec 15;6:1096. doi: 10.3389/fpls.2015.01096 (PMC4678209; doi:10.3389/fpls.2015.01096)
Supplement: Supplementary file 1 [file Table_1.PDF]

Supplementary Table 1: Mean comparison between the 27 segregants from the IL789 'immortalized F2'.

Genotype[a] (7/8/9)													BXY_Least Sq Mean [b]	Δ% from L/L/L [c]
P/H/P	A												759	145%
P/H/H	A												720	132%
L/H/P	A	B											696	125%
H/H/P	A	B	C										630	103%
H/H/H		B	C	D									574	85%
P/P/P		B	C	D	E								574	85%
P/L/P			C	D	E	F							539	74%
L/H/H			C	D	E	F							538	73%
L/L/P			C	D	E	F							536	73%
H/L/P			C	D	E	F	G						512	65%
H/P/P				D	E	F	G	H					491	58%
P/P/H				D	E	F	G	H	I				467	51%
L/L/H				D	E	F	G	H	I				465	50%
P/L/H				D	E	F	G	H	I	J			457	47%
L/H/L				D	E	F	G	H	I				456	47%
H/L/H					E	F	G	H	I	J			451	46%
H/H/L						F	G	H	I	J			444	43%
H/L/L						F	G	H	I	J	K		430	39%
P/H/L						F	G	H	I	J	K		423	36%
H/P/H						F	G	H	I	J	K		415	34%
H/P/L							G	H	I	J	K		396	28%
P/L/L								H	I	J	K		376	21%
L/P/P									I	J	K		349	13%
L/P/H									I	J	K		347	12%
P/P/L										J	K		330	7%
**L/L/L**											**K**	**L**	310	0%
L/P/L												L	200	-35%

[a] genotypic description of the 27 segregants at the three introgressions. Each letter represents the genotype at an introgression in the following order: IL7-5-5/IL8-3/IL9-2-5. L=homozygote *lycopersicum*, H=Heterozygote, P=homozygote *pennellii*. [b] Brix*Yield (BXY; g sugar/m^2). [c] Effects are expressed as percent difference from the nearly-isogenic internal reference line that is homozygous *lycopersicum* (L/L/L) at the three QTLs and was not statistically different from M82. Genotypes that don't share any letters are significantly different (at p<0.05).
