# Supplementary material for: Mendelizing all Components of a Pyramid of Three Yield QTL in Tomato
Source: Front Plant Sci. 2015 Dec 15;6:1096. doi: 10.3389/fpls.2015.01096 (PMC4678209; doi:10.3389/fpls.2015.01096)
Supplement: Supplementary file 2 [file Data_Sheet_1.DOCX]

**Supplementary Figure 1: Comparison between introgression and linkage mapping approaches**

Graphical illustration of the differences between introgression and linkage mapping. Both mapping approaches start from a cross between diverse inbred lines that provide phenotypic diversity and genetic polymorphism. Nearly-isogenic introgression lines (ILs, left side) are developed through several consecutive cycles of backcrossing followed by genome-wide, marker-assisted selection for presence of a single marker-defined introgressed segment per line and for validation of complete coverage (with overlaps) of the donor genome across the population. This step is followed by several selfing generations to create fixed inbreds that can be used as a permanent mapping resource. Whole-genome segregating populations for linkage-mapping are generated by fixing the F2 segregation derived from the initial cross. The fixation is achieved through inbreeding or through creation of double-haploids. The result is a segregating population of inbred lines where on average each line contain 50% of the genome from each of the parental lines. Using ILs for mapping main effects through QTL mapping is based on the comparison of each of the lines in the population to the common recurrent parent. Due to the nearly-isogenic nature of the IL population, any significant phenotypic difference between an IL and the recurrent parent is mapped to the defined genomic interval of the introgression. This population structure provides enhanced statistical power for detection of main-effect QTLs. QTL mapping via linkage mapping (right side) is based on the comparison of the phenotypic mean between all lines possessing allele A at a given marker and all lines possessing allele B at the same marker. Such a comparison is performed for all the markers that were used to genotype the population. A QTL is defined in a region where a significant difference is found between the genotypic classes for a marker or several linked markers. The main difference between these approaches for main-effect QTL mapping lies in the fact that in IL mapping, the tested interval is analyzed under a uniform background and compared to a defined reference line, while in linkage mapping, the effect of a QTL (or a marker) is tested in a segregating background and the allelic effect is an averaged value that is compared to a pool of diverse lines. These differences between the main-effect mapping approaches cascade also to the testing of genetic interactions (epistasis). Digenic interactions can be tested in linkage mapping populations as part of the mapping statistical procedure. The rationale is similar and is based on the comparison between the effect of lines containing a marker-defined digenic combination and the sum of the individual effects of lines containing contrasting alleles at each marker. Significant deviation of the digenic combination effect from the sum of independent effects implies epistasis. Testing of higher order interactions in common linkage mapping populations is not effective due to the rarity of these combinations and the lack of statistical power. When using an IL population, interactions are tested separately from main-effect mapping. Digenic interactions are tested through directed crosses of the target ILs to create double ILs. Epistasis is tested through the comparison between the effect of double ILs and the sum of the single IL effects. While the analysis of epistasis using IL population requires further crosses, the nearly-isogenic targeted approach provides increased statistical power.
