# Supplementary figures and images for: Mendelizing all Components of a Pyramid of Three Yield QTL in Tomato
Source: Front Plant Sci. 2015 Dec 15;6:1096. doi: 10.3389/fpls.2015.01096 (PMC4678209; doi:10.3389/fpls.2015.01096)

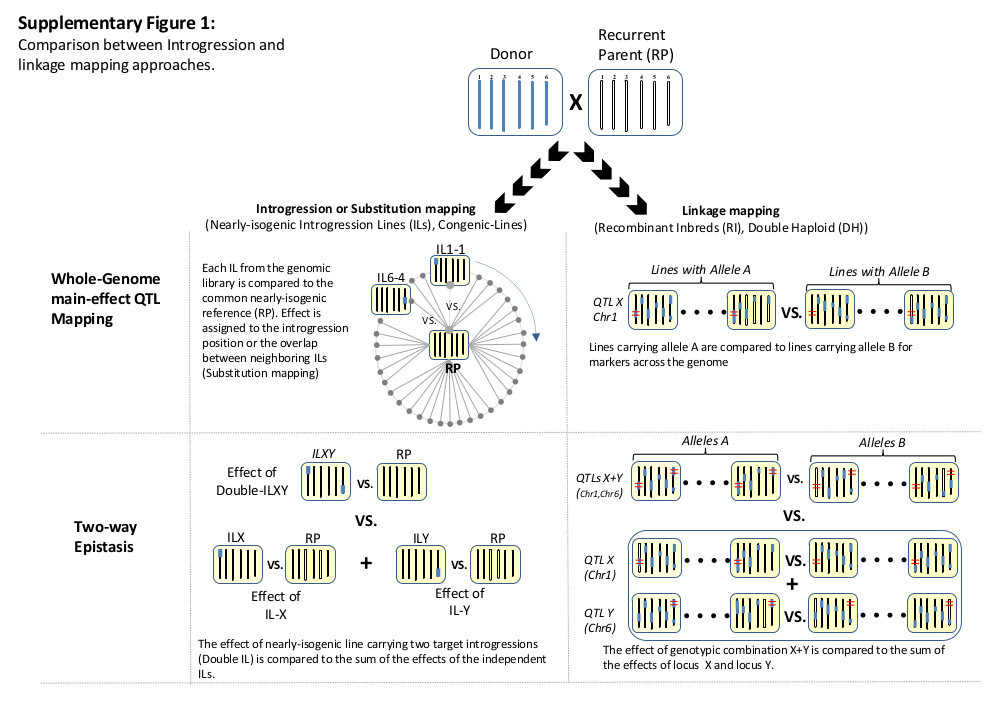

Supplement: Supplementary file 3 [file Image_1.TIFF]
